# Serum folate concentration and health-related quality of life among the elderly in South Korea

**DOI:** 10.1186/s12955-021-01899-2

**Published:** 2021-12-20

**Authors:** Eunmi Lee, Sangshin Park

**Affiliations:** Graduate School of Urban Public Health & Department of Urban Big Data Convergence, University of Seoul, 163 Seoulsiripdae-ro, Dongdaemun-gu, Seoul, 02504 Republic of Korea

**Keywords:** Folate, Folic acid, Health-related quality of life, Elderly

## Abstract

**Background:**

The purpose of this study was to investigate the association between serum folate concentration and health-related quality of life (HRQOL) among the elderly in South Korea.

**Materials and methods:**

The data used in this study were drawn from 1,021 participants over 65 years old in the Korea National Health and Nutrition Examination Survey from 2016–2018. HRQOL was measured by the EQ-5D questionnaire. Participants were divided into tertiles of folate concentration (ranges 1.7–5.6, 5.7–9.4, and 9.5–31.9 ng/mL). We performed multivariable linear regression to examine the relationship between folate and HRQOL, and multivariable logistic regression to examine the relationship between folate and the dimensional problem of HRQOL.

**Results:**

Higher folate concentrations were significantly associated with higher HRQOL in the elderly. The average HRQOL score of the elderly in the highest tertile of the folate level was 0.0289 higher than that of the lowest tertile (coefficient: 0.0289; 95% CI 0.0016, 0.0563). The HRQOL score increased by 0.0174 points when the folate concentration increased by 100%. When analyzing specific dimensions, a significant association with folate concentration was found only for the self-care dimension of HRQOL (odds ratio for self-care problems: 0.63; 95% CI 0.41, 0.99).

**Conclusions:**

The elderly with higher serum folate concentration tended to have higher HRQOL. Among HRQOL dimensions, self-care was only significantly associated with folate concentration.

## Introduction

Recently, an increasing number of studies have focused on health-related quality of life (HRQOL) as its importance has grown [1–3]. Testa et al. [4] and McDonald et al. [5] proposed that HRQOL is a multidimensional concept that includes areas related to physical, mental, emotional and social functions. The authors of the latter study also emphasized that HRQOL focuses on the impact of an individual's health status on their quality of life [5]. The fact that HRQOL is affected by diseases such as obesity [6], hypertension [3, 7], and diabetes [8] has already been proven through multiple studies. As the importance of HRQOL has increased, the development of HRQOL has become one of the primary purposes of public health research [9].

The EQ-5D is one of the most used HRQOL measurement tools. The EQ-5D consists of five dimensions (mobility, self-care, usual activities, pain/discomfort, and anxiety/depression). The EQ-5D-3L is designed to let participants choose one of the three levels (e.g., no, some, or severe problem) based on the severity of each dimension. The EQ-5D-5L is an updated version of the EQ-5D-3L which includes five severity levels (e.g., no problems to extreme problems/unable to). The EQ-5D-Y assesses HRQOL of children and adolescents. Of each version of EQ-5D, the EQ-VAS assesses self-rated health on a vertical scale from zero to 100, which means worst to best health imaginable. In the EQ-5D, severity levels in five dimensions are converted into a single score by applying a formula [10].

The nutrition status of the elderly is of grave importance as it is related to morbidity and mortality in the elderly [11]. The effects of nutritional status on physical and psychological well-being are greater in the elderly than in other age groups [12]. A particularly important factor is folate nutrition, which is associated with various important health problems in the elderly, such as cardiovascular disease, cancer, and cognitive function [11]. Accordingly, folate insufficiency contributes to decreased HRQOL in the elderly [13].

Folate is a water-soluble vitamin that is related to the other B vitamins, and it is a key nutritional factor in general cell growth and replication [14]. The intake recommendation of folate varies by age, sex, and individual health condition, but the typical recommendation for all aged 14 or older is 400 µg per day [15, 16]. Appropriate folate intake facilitates cell division and homeostasis, which is the essential role of folate coenzymes in nucleic acid synthesis, as well as methionine regeneration; in the shuttling, the oxidation and reduction of one-carbon units is required for normal metabolism and regulation [17]. On the other hand, inappropriate intake or malabsorption of folate may lead to harmful consequences, such as increased risk of chronic diseases and developmental disorders [17].

Numerous studies have been published on the relationship between folate levels and chronic diseases [18, 19]. For example, patients with rheumatoid arthritis had decreased cardiovascular mortality risks when their serum folate levels were at least 4.3 ng/mL [18]. Lower serum folate levels were associated with higher all-cause, cardiovascular, and cancer mortalities [19]. However, only few studies reported the relationship between folate levels and chronic diseases or conditions in South Korea [20, 21]. For example, the Korean elderly whose serum folate levels were declined from > 8.2 ng/mL to ≤ 5.9 ng/mL had 2.4 times higher risks for dementia than those with maintaining serum folate levels > 8.2 ng/mL during the 4-year follow-up period [20]. Another study showed the negative association between serum folate levels and body mass index (BMI) at mid- and late pregnancy in Korea [21].

Aging is one of the major causes of decreasing HRQOL, specifically due to factors such as biological senility and socio-psychological variations [22]. Diseases such as Alzheimer's disease and rheumatoid arthritis, which are particularly common in the elderly, have been proven in many studies to significantly decrease the HRQOL of the individuals with such diseases [23, 24]. These diseases reduce HRQOL by causing physical discomfort, while depression reduces HRQOL due to the associated mental and emotional problems [25]. Although many studies have examined the relationship between folate and various diseases that significantly reduce HRQOL [26–30], no studies have investigated the relationship between folate and HRQOL.

As we have seen, HRQOL is increasingly becoming important in relation to the health of an individual. Based on the existing research on the relationship between folate and various diseases, we conducted this study beginning with the hypothesis that a higher folate concentration is associated with a higher HRQOL. Therefore, this study was performed to examine the relationship between folate and HRQOL among the elderly using national representative sampling data.

## Methods

### Study area and design

This study was performed using the data obtained from the Korea National Health and Nutrition Examination Survey (KNHANES) conducted from 2016 to 2018 (https://knhanes.cdc.go.kr/knhanes/sub03/sub03_02_05.do). The KNHANES has been conducted by the Korean Centers for Disease Control and Prevention (KCDC) since 1998 to evaluate the health and nutritional status of Koreans. The survey has been widely accepted for its efficiency for use in statistical analyses of the health and nutrition status of Korean citizens.

The KNHANES consists of three surveys: a health examination, a health interview, and a nutrition survey. The health examination includes body measurement, blood pressure and pulse measurement, blood and urine test, oral examination, pulmonary function test, eye test, and grip test. The health interview consists of household and individual interviews. The household interview is administered to one adult (over 19 years old) of the household to assess the number of household members, household types, and household income. The individual interview is administered to each member of the household to assess morbidity, healthcare utilization, activity restriction, education and economic activities, physical activities, as well as smoking, drinking, mental health, safety consciousness, and oral health. The nutrition survey is conducted to evaluate the status of dietary behavior, dietary supplements, nutritional knowledge, food stability, and the contents of food intake one day before the survey.

### Sampling

The target population of the KNHANES was extracted using the two-step stratified cluster sampling, so that the representativeness of the Korean population could be obtained. Participants of KNHANES were drawn from 576 locations and approximately 13,000 households in each wave over the 3-year period (i.e., 192 locations and 3,840 households annually). For sampling, KNHANES stratified Korean population according to city/province (Seoul, Busan, Daegu, Incheon, Gwangju, Daejeon, Ulsan, Sejong, Gyeonggi, Gangwon, Chungbuk, Chungnam, Jeonbuk, Jeonnam, Gyeongbuk, Gyeongnam, and Jeju), dong/eup/myeon, and home types. They considered ratios of residential area and education level of head of household for sampling. Of 24,269 participants of KNHANES 2016-2018, our study used the data of 1021 participants who were aged 65 years or older, who completed the EQ-5D questionnaire, and who were measured for serum folate levels.

### Data collection instrument, process, and management

Blood samples were collected in the morning with overnight fast. Serum folate levels, our independent variable of interest, were measured through Chemiluminescent Microparticle Immunoassay (ARCHITECT i4000Sr; Abbott, Abbott Park, IL).

KNHANES measured HRQOL, our dependent variable of interest, using EQ-5D questionnaire. The elderly was surveyed on EQ-5D through face-to-face interviews. The EQ-5D consists of five dimensions (mobility, self-care, usual activities, pain/discomfort, and anxiety/depression). Each dimension has three health status levels meaning no problem, some problem, and a severe problem. The EQ-5D score was derived by combining all scores for the five dimensions, which ranged from 0 to 1; 0 indicating death and 1 indicating perfect health state. The score less than 0 means health state worse than death. The EQ-5D score was estimated through N3 model [31].

### Operational definitions

Marital status was divided into two categories: ‘married and living together’ and the others. Education level was divided into three categories: ≤ middle school, high school, and ≥ college. Employment status was divided into two categories: employed and unemployed. Household income was divided into quartiles: low-, lower-middle-, upper-middle-, and high-income groups. A current smoker was defined as a person who had smoked more than five packs of cigarettes and who were currently smoking. An alcohol consumer was defined as a person who had drunk at least once a month within a year. Performing aerobic physical activity was defined as moderate aerobic physical activity ≥ 150 min/week or intense aerobic physical activity ≥ 75 min/week.

BMI was calculated as body mass divided by the square of the body height, with body mass in kilograms and body height in meters. Diabetes mellitus was defined as fasting blood sugar ≥ 126 mg/dL, taking a medicine/insulin, or having been diagnosed by a doctor. Hypertension was defined as systolic blood pressure of 140 mmHg or higher, diastolic blood pressure of 90 mmHg or higher, or taking a relevant medicine. An individual was classified as having dyslipidemia if he or she met any of the following criteria: fasting triglyceride ≥ 200 mg/dL, high-density lipoprotein cholesterol < 40 mg/dL, total cholesterol ≥ 240 mg/dL, or taking any dyslipidemia medicine.

### Data entry, analysis, and interpretation

All analyses were conducted using SAS software version 9.4 (SAS Institute Inc., Cary, NC, USA) while accounting for the sampling design of complex surveys. The independent and dependent variables of interest were serum folate levels and HRQOL, respectively. We performed multivariable linear regression to analyze the relationship between folate levels and HRQOL. Model 1 examined the relationship between folate levels and HRQOL without adjustment. In model 2, we adjusted for age and sex; in model 3, we further adjusted for marital status, education level, employment status, household income, current smoking, alcohol consumption, aerobic physical activity, and BMI; and in model 4, we further adjusted for diabetes mellitus, hypertension, and dyslipidemia.

We also performed multivariable logistic regression analysis to evaluate the relationship between serum folate levels and the dimensional problem (some or severe problem) of EQ-5D. Throughout the analysis, the p value < 0.05 was regarded as statistically significant. The folate level values were log-transformed. We then used log-transformed folate levels as independent variables in both linear and logistic regression models.

## Results

The mean age of the participants in this study was 72.5 years (range 65–80 years). The participants in each folate level group of T1 (1.7–5.6), T2 (5.7–9.4), and T3 (9.5–31.9) represented 33.2% (n = 339), 34.1% (n = 348), and 32.7% (n = 334) of all subjects, respectively (Table 1). The mean ages of the participants in T1, T2, and T3 were 73.2, 72.4, and 72.7 years, respectively. As the folate level increased, the percentage of females, participation in aerobic physical activity, pain/discomfort problems, and HRQOL increased, while the percentage of marital status (married and living together), current smoking, alcohol consumption, diabetes mellitus, mobility, and self-care problems decreased. The increase in folate levels was not significantly associated with age, education level, employment status, household income, BMI, hypertension, dyslipidemia, usual activities, or anxiety/depression problems.

**Table 1:** Basic characteristics of the study participants by folate level

Characteristic	Tertiles of folate level (range, ng/mL)			*P*
	T1 (4.3, 1.7–5.6)	T2 (7.1, 5.7–9.4)	T3 (12.4, 9.5–31.9)	
Participants, n	339	348	334	
Age, years	73.2 (0.4)	72.4 (0.3)	72.7 (0.3)	0.20
Female, %	38.3 (3.1)	53.9 (3.0)	67.2 (2.8)	< 0.001
Marital status (married & living together), %	66.5 (3.0)	66.3 (3.1)	61.3 (3.3)	0.40
*Socioeconomic status variables*				
Education level, %				0.039
≤ Middle school	70.1 (3.0)	73.3 (2.8)	64.7 (3.3)	
High school	19.6 (2.5)	18.9 (2.5)	18.9 (2.6)	
≥ College	10.4 (2.0)	7.7 (1.6)	16.5 (2.3)	
Employment status, %				0.002
Employed	29.7 (2.9)	37.8 (3.2)	23.5 (2.6)	
Unemployed	70.3 (2.9)	62.2 (3.2)	76.5 (2.6)	
Household income, %				0.12
Low	47.2 (3.5)	46.4 (3.4)	43.8 (3.3)	
Lower middle	27.9 (3.1)	27.6 (3.0)	23.1 (2.5)	
Upper middle	17.6 (2.8)	16.1 (2.5)	17.5 (2.5)	
High	7.3 (1.6)	9.9 (1.9)	15.7 (2.4)	
Lifestyle variables				
Current smoking, %	15.5 (2.2)	6.7 (1.7)	4.9 (1.3)	< 0.001
Alcohol consumption (≥ 1 drink/month), %	42.6 (3.2)	36.6 (2.9)	26.8 (2.6)	0.001
Aerobic physical activity, %	27.1 (2.8)	32.5 (2.9)	37.4 (3.1)	0.040
Disease variables				
BMI, mean	23.9 (0.2)	24.2 (0.2)	24.0 (0.2)	0.53
Diabetes mellitus, %	30.2 (3.2)	26.4 (2.7)	23.8 (2.6)	0.30
Hypertension, %	68.1 (2.7)	62.2 (3.2)	62.8 (3.0)	0.30
Dyslipidemia, %	55.1 (3.2)	54.3 (3.0)	57.7 (3.1)	0.73
HRQOL (score of EQ-5D)	0.866 (0.01)	0.877 (0.01)	0.887 (0.01)	0.30
Problem in dimensions, %				
Mobility	40.4 (3.2)	37.2 (3.3)	34.7 (3.2)	0.44
Self-care	14.2 (2.2)	10.2 (1.9)	8.5 (1.6)	0.09
Usual activities	22.3 (2.4)	21.7 (2.9)	22.2 (2.6)	1.00
Pain/discomfort	36.9 (3.2)	37.0 (3.2)	38.4 (3.0)	0.94
Anxiety/depression	15.8 (2.5)	17.6 (2.4)	15.2 (2.0)	0.74

**Fig. 1:**
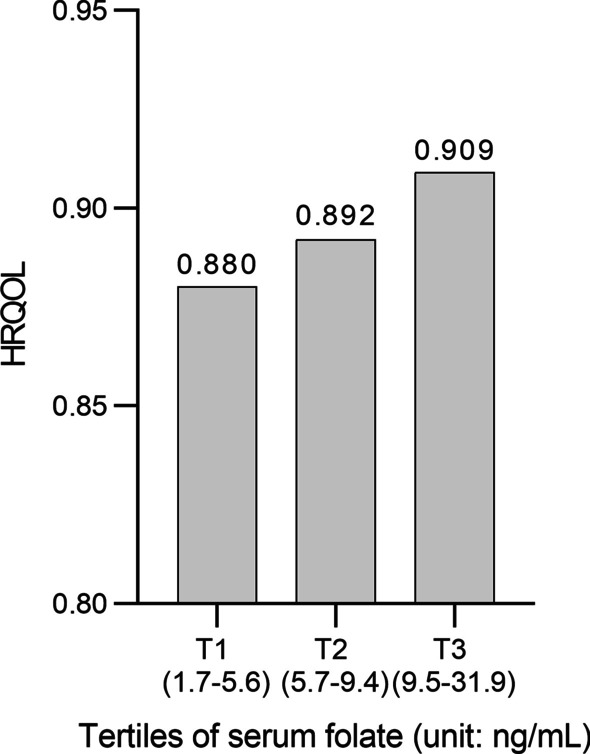
The HRQOL scores by folate level after adjusting for age, sex, marital status, education level, employment status, household income, smoking, alcohol consumption, aerobic physical activity, BMI, diabetes mellitus, hypertension, and dyslipidemia

The HRQOL scores were 0.880 (T1), 0.892 (T2), and 0.909 (T3) at each level of folate concentration after adjusting for all confounding variables (Fig. 1).

In adjusted models, HRQOL was also significantly associated with folate concentration (Table 2). After adjusting for age and sex, the HRQOL score of T3 was 0.0399 higher than that of T1 [Model 2; coefficient: 0.0399; 95% confidence interval (CI) 0.0122, 0.0676; p for trend: 0.005]. For Model 4, diabetes mellitus, hypertension, and dyslipidemia were added to Model 3 to test the outcomes after controlling for all variables. As a result, the HRQOL score of T3 was 0.0289 higher than that of T1 [Model 4; coefficient: 0.0289; 95% CI 0.0016, 0.0563; p for trend: 0.038].

**Table 2:** Coefficient (95% CI) of HRQOL by folate concentration

	Tertiles of folate level (median, range, ng/mL)			*P* for trend
	T1 (4.3, 1.7–5.6)	T2 (7.1, 5.7–9.4)	T3 (12.4, 9.5–31.9)	
Participants, n	339	348	334	
Model 1	Reference	0.0117 (-0.0195, 0.0428)	0.0219 (-0.0060, 0.0499)	0.12
Model 2	Reference	0.0177 (-0.0103, 0.0457)	0.0399 (0.0122, 0.0676)	0.005
Model 3	Reference	0.0120 (-0.0148, 0.0389)	0.0295 (0.0021, 0.0569)	0.035
Model 4	Reference	0.0118 (-0.0148, 0.0385)	0.0289 (0.0016, 0.0563)	0.038

Model 1: not adjusted; Model 2: adjusted for age and sex; Model 3: further adjusted for marital status, education level, employment status, household income, smoking, alcohol consumption, aerobic physical activity, and BMI; Model 4: further adjusted for diabetes mellitus, hypertension, and dyslipidemia

A significant relationship between folate and HRQOL was found when analyzing folate concentration as a continuous variable [coefficient: 0.0251; 95% CI 0.0018, 0.0483; p value: 0.035] (Table 3). When the folate concentration increased by 100%, the HRQOL score increased by 0.0174 points (coefficient 0.0251 × ln (2) = 0.0174).

**Table 3:** Coefficient (95% CI) of HRQOL and folate concentration

Model	Coefficient (95% CI)	P value
1	0.0202 (-0.0026, 0.0429)	0.082
2	0.0338 (0.0104, 0.0571)	0.005
3	0.0253 (0.0021, 0.0485)	0.033
4	0.0251 (0.0018, 0.0483)	0.035

Model 1: not adjusted; Model 2: adjusted for age and sex; Model 3: further adjusted for marital status, education level, employment status, household income, smoking, alcohol consumption, aerobic physical activity, and BMI; Model 4: further adjusted for diabetes mellitus, hypertension, and dyslipidemia

When HRQOL was examined according to each dimension, folate concentration was particularly related to self-care problems (Table 4). The odds ratio of self-care problems and folate concentration in Model 4 is 0.63 (OR 0.63; 95% CI 0.41, 0.99). When the folate concentration increased by 100%, the self-care problem decreased by approximately 27% (exp (ln (0.63) × ln (2)) = 0.73).

**Table 4:** Odds ratios (95% CI) of HRQOL dimensional problems and folate concentration

Model	Mobility	Self-care	Usual activities	Pain/discomfort	Anxiety/depression
1	0.83 (0.62, 1.11)	0.58 (0.39, 0.85)**	0.97 (0.71, 1.32)	1.02 (0.76, 1.39)	0.97 (0.68, 1.40)
2	0.71 (0.51, 0.99)*	0.55 (0.37, 0.84)**	0.87 (0.62, 1.22)	0.81 (0.58, 1.13)	0.88 (0.61, 1.28)
3	0.80 (0.56, 1.14)	0.63 (0.40, 0.98)*	0.99 (0.69, 1.44)	0.88 (0.62, 1.24)	0.87 (0.59, 1.29)
4	0.79 (0.56, 1.13)	0.63 (0.41, 0.99)*	1.00 (0.69, 1.45)	0.88 (0.62, 1.24)	0.88 (0.59, 1.30)

Model 1: not adjusted; Model 2: adjusted for age and sex; Model 3: further adjusted for marital status, education level, employment status, household income, smoking, alcohol consumption, aerobic physical activity, and BMI; Model 4: further adjusted for diabetes mellitus, hypertension, and dyslipidemia. *p < 0.05, **p < 0.01, ***p < 0.001

## Discussion

To date, no study has examined the relationship between folate and HRQOL. This study showed that folate and HRQOL are related; specifically, the higher serum folate concentration has the higher HRQOL. The increase in the score of HRQOL as folate concentration increased was significant in the model that adjusted several variables. Among the EQ-5D dimensions, folate concentration was most related to self-care problems. As folate concentration increased, self-care problems significantly decreased.

Many studies have demonstrated relationships between folate and various diseases, such as obesity [32], hypertension [33], and diabetes [34]. In addition, several studies have already proven that such chronic diseases are associated with a decrease in HRQOL [3, 6–8]. Thus, we were interested in the relationship between folate and HRQOL among the elderly, and we had a strong expectation that there would be a positive relationship between folate and HRQOL.

The mechanisms governing the relationship between folate intake and HRQOL are unclear. However, considering that studies have shown that high folate intake lowers the incidence of various diseases and that patients with such diseases have lower HRQOL, it can be expected that increased serum folate levels may improve HRQOL.

Several studies have supported the hypothesis that folate intake could act as a protective factor against obesity [32, 35, 36]. Further, studies have shown significant associations between serum folate, DNA methylation, BMI, body fat ratio [37], and high folate intake from fortified foods with high DNA methylation [35, 38]. The methionine cycle is a metabolic process by which methionine becomes homocysteine via S-Adenosylhomocystein and S-Adenosylmethionine, and then homocysteine is converted back into methionine by recovering the methyl group from the folate cycle [39]. The types of nutrition deficiency that can lead to damage to homocysteine metabolism include folate deficiency, which increases the concentration of plasma homocysteine [40]. As a result, high homocysteine may serve as a risk factor for obesity [41]. Therefore, adequate levels of folate in the blood do not increase homocysteine levels, which may help prevent obesity.

Similarly, it is speculated that adequate levels of folate in the blood may lower risks of hypertension by lowering the homocysteine levels. Hyperhomocysteinemia is responsible for hypertension by increasing oxidative stress [42–45]. Increased homocysteine in plasma causes oxidative stress and endothelial dysfunction, leading to vasoconstriction, vasodilation by nitric acid, and nitrate reduction, all of which increase blood pressure [42, 44]. A high homocysteine level, also called hyperhomocysteinemia, is known to be regulated through the intake of grain fortification foods containing folate [46] and folic acid supplements [47]. In a recent meta-analysis study of randomized experiments, folate intake has been proven to be effective in reducing blood pressure and homocysteine levels among patients with hypertension and hyperhomocysteinemia [42, 48].

Our study showed that serum folate concentration was associated with self-care problems. Low folate concentration was likely to affect self-care problems through sarcopenia. Previous studies showed the relationship between low intakes of folate and the risk of sarcopenia [49] and the relationship between the sarcopenia and the impairment in self-care [50].

This is the first study to examine the specific relationship between folate and HRQOL. While there have been studies investigating the relationship between folate and diseases [32–34] or diseases and HRQOL [3, 6–8], there have been no studies examining the relationship between folate and HRQOL. Another strength is that this study was conducted using national representative sample data. These data were obtained from KNHANES, which indicates that the study participants are representative of the Korean population. Therefore, the results of this study can be generalized to the entire population. However, because the results of this study rely on the cross-sectional study of KNHANES, it has a limitation in that it cannot clearly elucidate the causal relationship between folate and HRQOL.

## Conclusions

Increased levels of serum folate were associated with increased HRQOL and decreased problems in self-care dimension of HRQOL in the Korean elderly. Our findings suggest that maintaining adequate levels of serum folate is possibly helpful in improving HRQOL in the elderly. Further prospective studies are required to confirm the causality that increased levels of serum folate improves HRQOL.

## Data Availability

The datasets are available in the Korea Centers for Disease Control and Prevention (https://knhanes.cdc.go.kr/knhanes).
